# The relationship between parents' oral hygiene knowledge and children with Down Syndrome's oral hygiene via OHI-S

**DOI:** 10.12688/f1000research.87848.1

**Published:** 2022-03-31

**Authors:** Agung Sosiawan, Dian Agustin Wahjuningrum, Dini Setyowati, Michelle Suhartono, Natasha Winona Audrey, Tata Prasantat Mawantari, Fery Setiawan, Ajinkya M. Pawar

**Affiliations:** 1 Department of Dental Public Health, Faculty of Dental Medicine, Universitas Airlangga, Surabaya, Indonesia; 2 Department of Odontology Forensic, Faculty of Dental Medicine, Unversitas Airlangga, Surabaya, Indonesia; 3 Department of Conservative Dentistry, Faculty of Dental Medicine, Universitas Airlangga, Surabaya, Indonesia; 4 Faculty of Dental Medicine, Universitas Airlangga, Surabaya, Indonesia; 5 Doctoral Program of Medical Science, Faculty of Medicine, Universitas Airlangga, Surabaya, Indonesia; 6 Department of Conservative Dentistry and Endoodntics, Nair Hospital Dental College, Mumbai, Maharashtra, 400008, India

**Keywords:** Primary care, Mental Disease, Down Syndrome, Index Score OHI-S, Oral Hygiene

## Abstract

**Background:** Down Syndrome (DS) is a genetic disorder responsible for mental and development retardation. DS occurs when a person has an extra chromosome (47 instead of 46), the third copy of the Trisomy 21 chromosome. This causes structural and functional anomalies in the human body and some degree of intellectual disability. Children with DS have poor oral hygiene as they are unable to understand and are unaware of its importance. Oral hygiene problems commonly found in children with DS are gingivitis, periodontal problems, pain, infection, and problems with the masticatory system. This study explored the relationship between parents' knowledge and maintenance of the oral hygiene of children with DS through the OHI-S (Oral Hygiene Index Simplified) Index Score.

**Method:** This study was conducted by distributing 25 questions via Google Form to 100 subjects that belong to POTADS (Down Syndrome Parents Association. The participants were children diagnosed with Down Syndrome and their parents. Questionnaires were used to assess parents' knowledge about their child's oral hygiene. To assess the children’s oral hygiene, dental exams were performed with the OHI-S on six tooth surfaces. To calculate the OHI-S score for each individual, the debris and calculus scores from the 6 surfaces of the teeth were totalled and divided by six.

**Results:** The relationship between parents' knowledge and the maintenance of oral hygiene of children with DS was found by conducting a linear regression analysis and hypothetical test. The equation of Y = 77.734 + (-7.377) X was achieved through linear regression analysis, and indicated that a 1% increase in parents' knowledge caused a reduction in OHI-S score to 7.377. The hypothetical test showed that parents' knowledge affected their children's OHI-S score significantly.

**Conclusion:** There was a significant contradictive relationship between parents' knowledge and the maintenance of oral hygiene of children with DS **.**

## Introduction

Children with special needs require more care than those without. Special care is given to these children as they carry specific physical, mental, intellectual, social, and emotional disabilities or abnormalities. ^1^ Mentally disabled children are one of many examples of special needs children as they possess a below-average intellect with mild, severe, and very severe abnormalities (IQ between 25 and 70). ^2^

Down Syndrome or DS is a genetic disorder that affects a child's development and causes mental retardation. Down Syndrome occurs when a person has a third copy of the Trisomy 21 Chromosome. The third copy of Trisomy 21 causes structural and functional anomalies in the human body and some degree of intellectual disability. ^3^ Down Syndrome occurs in one of 700 births and is present in all ethnicities. The chance of someone having a baby with DS is higher when she is 35 years old or older, and baby boys are more likely to have DS rather than baby girls. ^4^ Children with DS tend to have poor oral hygiene as they are unable to understand and are unaware of the importance of oral hygiene. ^5^ Oral hygiene problems commonly found in children with DS are gingivitis, periodontal problems, pain, infection, and problems with the masticatory system. ^6^ Mental retardation or MR, developmental delay, physical disability, and other problems could affect disabled children's daily activities and are reasons why they could not maintain their oral hygiene. Therefore, it is necessary for parents, nannies, and people close to these children to help them maintain their oral hygiene. ^7^

Children's oral hygiene has become a particular concern in this era. Knowledge is the result of curiosity and occurs because someone senses particular objects through human senses. Education regarding children's oral hygiene should be obligatory for parents as it could significantly help their children's teeth development and growth. The parents’ knowledge and ability in maintaining their children's oral hygiene could be influenced by several aspects such as age, education, economic and social status, experience, mass media, and environment. The parents' ability to maintain their children's health will significantly impact their children’s attitude and behavior. ^8^

To boost awareness and effort towards the maintenance of the oral hygiene of children with special needs, this study analyzed parents' knowledge levels in maintaining their children's oral hygiene at POTADS (Down Syndrome Parents Association) Surabaya.

## Methods

### Ethical statement

We obtained ethical approval for this study from Universitas Airlangga Faculty of Dental Medicine Health Research Ethical Clearance Commission on 21-2-2020 (058/HRECC.FODM/II/2020). Due to the COVID-19 pandemic, ethical approval was also re-issued on 24-02-2021 (082/HRECC.FODM/II/2021).

Written informed consent was obtained from the parents for their own and for their children’s involvement in this study as children were not able to consent. Participants were informed of their right to withdraw and that they could refuse to answer any questions, end the survey, or refuse the child’s dental exam at any point.

### Research design

An analytical-observational study was conducted in Surabaya, 2020-2021, via Google Forms to 100 parents whose children require special care (Down Syndrome) in POTADS Surabaya. The sample size was based on the Lemeshow formulas. The dental examination procedure is further explained below.

### Participants

The populations of this study are children who require special care (Down Syndrome) and their parents in POTADS Surabaya. Inclusion criteria are parents whose children were diagnosed with Down Syndrome, children aged 7-9 years old, and parents or guardians who agreed to be a part of this study. The samples used in this study were purposive as they were taken based on specific considerations of the researcher.

### Research procedure

The potential participants were recruited from the POTADS. We also used social media, such as Twitter, WhatsApp, Instagram, and Line, as our recruitment strategy to identify the POTADS’ members and distribute the questionnaire. To be eligible for participation in this study, the participants had to be parents whose children were aged 7 to 9 years old and were diagnosed with Down Syndrome, and be a POTADS’ member. Participants were first contacted to give their consent to participate. Those who did not consent did not progress to the questionnaire. The questionnaire had been tested for its validity and reliability.

We conducted a self-administered questionnaire survey using Google Forms to collect data about parents' knowledge. After the participants completed the questionnaire, we contacted them to arrange a time to do their child’s oral hygiene exams at the place most convenient to them. All participants who had completed the questionnaire had their child participate in the oral hygiene examinations. To assess their child’s oral hygiene, we used a basic dental diagnostic kit comprising two dental mirrors, one dental explorer, and one dental cotton tweezer. The principal researcher performed the child’s oral hygiene examinations using the oral hygiene index simplified (OHI-S). There were six tooth surfaces examined: the buccal surface of the upper right first molar (16), the labial surface of the upper right central incisor (11), the buccal surface of the upper left first molar (26), the lingual surface of the lower left first molar (36), the labial surface of the lower left central incisor (31), and the lingual surface of the lower right first molar (46). The OHI-S score consists of the Debris Index (DI) scores and the Calculus Index (CI) scores. The DI and CI scores represent the amount of debris and calculus, respectively, on the six tooth surfaces. To calculate the OHI-S score for each individual, the DI and CI scores were totalled and divided by six (the number of tooth surfaces examined). During the oral hygiene examination, the challenge we faced was the child’s resistant behaviors, such as pushing the hand or the dental instrument away, moving the head, and refusing to open their mouth. With the help of the parents, we made a few distractions to comfort the child and gain their cooperation.

Data were compiled in Excel 2016 before being added to SPSS v25.0 for analysis.

### Assessing children with down syndrome’s debris score via OHI-S

The debris score was assessed through the existence of debris found by using OHI-S. The score 0 was chosen if there was not any debris or stain, score 1 was chosen if soft debris or extrinsic stains were found covering one-third of a tooth surface, score 2 was chosen if soft debris was found covering more than one-third of a tooth surface but not more than two-thirds of a tooth surface, and score 3 was chosen if soft debris were found covering more than two-thirds of a tooth surface.

### Assessing children with down syndrome’s calculus score via OHI-S

The calculus score was assessed through the existence of calculus by using OHI-S. Score 0 was chosen if no calculus was found, score 1 was chosen if supragingival calculus were found covering not more than one-third of a tooth surface, score 2 was chosen if supragingival calculus or individual subgingival calculus spots were found to cover one-third but not more than two-thirds of a tooth surface, and score 3 was chosen if supragingival calculus was found covering more than two-thirds of a tooth surface.

### Assessing parents’ knowledge

Parents' knowledge was assessed through questionnaires that contained 25 questions (see extended data) and were distributed via social media. The ordinal scale was used to measure the parents' knowledge and was categorized as low and high. The low category was chosen if parents' knowledge levels were between 0-71.5, and the high category was chosen if parents' knowledge levels were between 72-100. We decided to use this level based on the median score as the cut-off point to categorize parents' knowledge.

### Statistical analysis

The collected data was analyzed using Statistical Package for the Social Science Software or SPSS (IBM SPSS Statistics 25.0). The questionnaire validity test searched for the correlation between individual questionnaire scores and the total score (bivariate). The reliability test was conducted using a reliability re-test, and the normality test was conducted using the Shapiro-Wilk method. The linear regression method was chosen if the data distribution was standard, and the ordinal regression method was chosen if the data distribution was not expected.

### STROBE cross sectional guidelines

We used STROBE's cross-sectional reporting guidelines to ensure research meets international standards for peer-reviewed articles. The checklist is completed by entering the page number of the manuscript where the reader can easily find each item listed. If we believe that an item is not valid, we will write “N/A” and provide a brief explanation in the STROBE cross-sectional reporting guidelines. ^9^

## Results

The characteristics of the samples were reported collectively with the findings of every factor, respectively (debris and calculus score via OHI-S and parents' knowledge level).

### Study subject characteristics

Study subject characteristics consisted of the gender, age, education, and profession of each subject. The subject characteristics are shown in Table 1.

**Table 1.** Gender, age, education, and profession of each subject.

Respondent characteristics		Frequency	Percentage
Gender	Male	20	20%
	Female	80	80%
Age	20-29 years old	20	20%
	30-39 years old	30	30%
	40-49 years old	10	10%
	50-59 years old	40	40%
Education	Elementary	10	10%
	Middle school	10	10%
	High school	70	70%
	College (diploma/bachelor)	10	10%
Profession	Entrepreneur	10	10%
	Teacher	25	25%
	Private sector employee	10	10%
	Housewife	55	55%

Based on Table 1, the parent participants were mostly female (80%). Respondents who participated in the study were mostly 30-39 and 50-59 years old (30% and 40% each respectively). The respondents of the study were primarily high school graduates (70%). Most of the respondents were housewives (55%).

### Relationship aspects of parents' knowledge level and calculus-debris score of children with down syndrome via OHI-S

Based on Table 2, it could be seen that 55.6% of all respondents possessed enough knowledge in maintaining children' oral hygiene, with a median score of ≥ 72. Scores below 72 are classed as low, meaning that the parents’ knowledge in maintaining children’s oral hygiene is low. Scores above 72 from the questionnaire are high, meaning that the parents’ knowledge is high.

**Table 2.** Parents; knowledge in maintaining children’s oral hygiene frequency.

Variable	Category	F	%
Knowledge	Low (0-71.5)	45	44.4%
	High (72-100)	55	55.6%
Total		100	100%

In Table 3, the frequency of OHI-S score of children with Down Syndrome (7-9 years old) in POTADS Surabaya could be seen. Children with Down Syndrome were mainly found to have good oral hygiene (65% of the total respondents). The average respondents were rated ≤ 1.2 on the OHI-S score.

**Table 3.** OHI-S score of children with down syndrome (7-9 years old) in POTADS Surabaya.

Variable	Category	F	%
OHI-S score	Good (0-1.2)	65	65%
	Moderate (1.3-3.0)	35	35%
	Poor (3.1-6.0)	0	0%
Total		100	100%

Based on Table 4, the average parents' knowledge and children with Down Syndrome’s OHI-S score could be seen (67.64 and 1.3683, respectively).

**Table 4.** The average parents’ knowledge level and children with down syndrome’s (7-9 years old) OHI-S score in POTADS.

Category	N	Mean
Parents’ Knowledge score	100	67.64
Children OHI-S score	100	1.3683

Table 5 exhibits the results of the simple regression analysis test. The test was conducted after the data significance (p > 0.05) was found by using the data normality test of Shapiro-Wilk. Furthermore, linearity, heteroscedasticity, and autocorrelation tests were also conducted, resulting in the simple regression analysis. The simple regression result between parents' knowledge toward OHI-S score was as follows:

Y=a+bx

$$Y=77.734+\left(-7.377\right)\;X$$

**Table 5.** Linear regression analysis on parents’ knowledge towards children’s OHI-S score.

Variable	Regression coefficient	T	Sig.
	77.734	148.321	0.000
Total	-7.377	-20.001	0.000

The interpretation of the linear equation above is as follows:

a) The A constant of 77.734 means that if the OHI-s score is equal to zero or constant, the value of knowledge consistency to the OHI-s score is 77.734.
b) The knowledge variable regression coefficient is – 20.001, meaning that if parents' knowledge increases by 1%, the OHI-S score will decrease by 20.001.
c) The significance value in Table 5 is 0.000 (p < 0.05), meaning that parental knowledge significantly affects children's OHI-S score.

Table 6 shows the data analysis value of R = 0.896 ^a^. The value indicates that the relationship between the dependent and independent variables is strong, as R > 0.5. The number of R square or the coefficient of determination is 0.803, which means that the independent variable could explain 0.803 or 80.3% of the variation of the dependent variable. At the same time, the remaining 19.7% is the result of other unexamined causes.

**Table 6.** Coefficient of determination.

R	R Square
0.896 ^a^	0.803

## Discussion

This study was conducted to understand the influence of parents' knowledge in maintaining children with special care’s oral hygiene in POTADS Surabaya. Parents' knowledge levels were obtained through an online survey which consisted of 25 questions about basic knowledge of oral hygiene for children with special care. The study's data found that the average parents' knowledge value was 67.64. Furthermore, the average OHI-S score of 7-9 years old children was 1.3683. This score indicates that they have well-maintained oral hygiene. ^10^

OHI-S measures a person's oral hygiene based on Debris Index (DI) and Calculus Index (CI). A person's oral hygiene could be measured by valuing the calculus and plaques. ^11^ The Sig. value of 0.000 was found as the result of this study. This value suggests that the parents' knowledge has significantly influenced children with special care’s oral hygiene in POTADS Surabaya. The hypothetical test was conducted by comparing the value of t-count with t-table, and the result was -20.001 of the t-table values. The value is greater than the t-count value, which is 2.365 (a negative value). If the value of t-count is more significant than t-table and is negative, it would be concluded that a contradictive influence existed in the relationship between parents' knowledge and children with special need's hygiene in POTADS Surabaya. The study conducted by Guswan and Yandi (2017) has proven that a higher level of parents' knowledge would mean a lower children's OHI-S score. ^12^

Knowledge is the foundation for the creation of action. A person is said to lack knowledge if he/she cannot recognize, explain, and analyze a situation. Proper knowledge could affect someone's action in improving health, especially in maintaining oral hygiene. On the other hand, a lack of knowledge could cause problems in maintaining oral hygiene, such as caries. ^12^ Parents are obligated to have a proper education in maintaining oral hygiene and should exhibit more concern in children's oral hygiene, especially if they need special care. Therefore, information regarding the significance of oral hygiene for parents is necessary. ^13^ Special training and adaptations for children with Down Syndrome are necessary to ensure that they brush their teeth effectively. This is due to their lack of motor skills compared to other children, and parents play a role in supervising and training children to brush their teeth effectively. ^14^

Moreover, as the response of Down Syndrome children to a given stimulus is much different from children without Down Syndrome in general, parents are required to be more creative and active when providing learning activities. ^15^ Based on the determinative coefficient data analysis result, it could be understood that the relationship between children with special needs’ OHI-S score and parents' knowledge level in maintaining oral hygiene is strong (R = 0.896 ^a^). The R square value of 0.803 or 80.3% means that the variation of the dependent variable, the OHI-S score of children with special needs, could be explained by the independent variable, namely the value of parents' knowledge about children's oral hygiene. Meanwhile, the rest were not investigated because we only focused on parents’ knowledge and the child’s oral hygiene.

The oral hygiene of children with Down Syndrome could be affected by various factors such as predisposing factors, enabling factors, and reinforcing factors. Predisposing factors are triggering factors or antecedents of behavior that provide reasons or motivation for the said behavior. ^16^^,^^17^ Enabling factors are factors that could facilitate behavior or actions conducted by individuals in pain. Furthermore, other factors included infrastructure and health facilities. ^18^ Resources were gathered from promotional media (booklet, leaflet, flyer, flipchart, and posters), electronic media (television, radio, video, slide, and filmstrip), and billboards. ^18^^,^^19^

Reinforcing factors could strengthen a person's motivation to change his/her action based on a rule or policy. ^20^ A family is one of many examples of reinforcing factors. In a family, various factors could influence health behavior in addition to parents' knowledge, such as parents' age, occupation, education, attitudes, and family support. ^21^ Parents whose children are diagnosed with Down Syndrome should have sufficient oral hygiene knowledge, such as appropriate usage of a toothbrush, appropriate nutritional provision, and appropriate methods in elevating children's psychomotor ability. Moreover, the effort to lift children with Down Syndrome's life quality through oral hygiene could avoid malnutrition and improve their nutritional intake and growth speed. ^13^ Therefore, it is important that parents' have sufficient oral hygiene knowledge as it could significantly influence their children's oral hygiene. Nevertheless, other factors could also affect children with Down Syndrome's oral hygiene.

One of the limitations of the study was that the self-administered questionnaire may cause bias in the participants’ responses. Another drawback was related to the selection of a place for the oral hygiene examination that was based on the parent's preference. Some of them did not have a good light to perform oral hygiene examination causing the potential bias during the examination.

## Conclusions

Contradictive influences were significant in the relationship between parents' knowledge about oral hygiene and children with special needs' oral hygiene in POTADS Surabaya. Further studies could be conducted to determine other factors that could affect children with special needs' oral hygiene.

## Data availability

### Underlying data

Mendeley: The Relationship Between Parents' Oral Hygiene Knowledge and Children with Down Syndrome's Oral Hygiene via OHI-S. https://doi.org/10.17632/329zrbpkc8.4. ^22^

This project contains the following files:

- Gender Age and Profession 100 Sampels.xlsx
- Raw Data Responses (Before and After COVID-19).xlsx

### Extended data

Mendeley: The Relationship Between Parents' Oral Hygiene Knowledge and Children with Down Syndrome's Oral Hygiene via OHI-S. https://doi.org/10.17632/329zrbpkc8.4. ^22^

This project contains the following files:

- List questionnaires.docx
- Table 1. xlsx
- Table 2. xlsx
- Table 3. xlsx
- Table 4. xlsx
- Table 5. xlsx
- Table 6. xlsx
- STROBE-checklist-v4-cross-sectional.doc

Data are available under the terms of the Creative Commons Attribution 4.0 International license (CC-BY 4.0).

## Author contributions

AS: designed, wrote, and made the firs design of manuscript, DAW: checked and involved in the correction of the manuscript, MS, NWA, TPM: helped AS in analyzing, conducting, and counting, the data DS: checked and helped AS in making, designing, and writing the whole manuscript, FS: helped AS in writing the manuscript, AMP: helped in submitting the manuscript.
