# Supplementary material for: The relationship between parents' oral hygiene knowledge and children with Down Syndrome's oral hygiene via OHI-S
Source: F1000Res. 2022 Apr 29;11:374. Originally published 2022 Mar 31. [Version 2] doi: 10.12688/f1000research.87848.2 (PMC9280112; doi:10.12688/f1000research.87848.2)
Supplement: Peer review history

# Peer review: Reviewer response for version 2

**DOI:** 10.5256/f1000research.133369.r138478

**Published:** 2022-07-13

**Authors:** Jumma Alkhabuli

**Affiliations:** 1 Oral Medicine, College of Dental Medicine, QU Health, Qatar University, Doha, Qatar

**Introduction:**

- Indonesia is a highly populated country and this reflects in the prevalence of Down syndrome. It would be proper to provide the prevalence of DS in the country. This also helps in determining the sample size.

**Methods:**

- Participants: “The samples used in this study were purposive as they were taken based on specific considerations of the researcher”. What is the specific consideration? Are they worth mentioning?
- Research procedure: Although a single examiner carried out the examination, I feel that intra-examiner consistency could have been performed!
- The examination kit should have included a head-light torch to ensure proper examination

**Results:**

- Table 3: “Children with Down Syndrome were mainly found to have good oral hygiene (65% of the total respondents)”. I think the word “respondents” is improper in this context. Because the % refers to the number of cases examined not responded to the questionnaire!
- Also, all abbreviations in the tables should be written in full as a footnote

**Discussion:**

- As mentioned, many factors could affect the outcome. Although it is feasible, wondering why parents’ education level for example was not correlated with the OH?

**General comment:**

- Academic language could have been improved

Is the work clearly and accurately presented and does it cite the current literature?

Partly

If applicable, is the statistical analysis and its interpretation appropriate?

Yes

Are all the source data underlying the results available to ensure full reproducibility?

Yes

Is the study design appropriate and is the work technically sound?

Partly

Are the conclusions drawn adequately supported by the results?

Partly

Are sufficient details of methods and analysis provided to allow replication by others?

Yes

Reviewer Expertise:

Oral biology, oral medicine, oral pathology

I confirm that I have read this submission and believe that I have an appropriate level of expertise to confirm that it is of an acceptable scientific standard.

# Peer review: Reviewer response for version 2

**DOI:** 10.5256/f1000research.133369.r136345

**Published:** 2022-05-03

**Authors:** Nor Azlida Mohd Nor

**Affiliations:** 1 Department of Community Oral Health & Clinical Prevention, Faculty of Dentistry, University of Malaya, Kuala Lumpur, Malaysia

Authors have addressed my previous comments.

Is the work clearly and accurately presented and does it cite the current literature?

Yes

If applicable, is the statistical analysis and its interpretation appropriate?

Partly

Are all the source data underlying the results available to ensure full reproducibility?

Yes

Is the study design appropriate and is the work technically sound?

Partly

Are the conclusions drawn adequately supported by the results?

Yes

Are sufficient details of methods and analysis provided to allow replication by others?

Partly

Reviewer Expertise:

dental public health

I confirm that I have read this submission and believe that I have an appropriate level of expertise to confirm that it is of an acceptable scientific standard.

# Peer review: Reviewer response for version 1

**DOI:** 10.5256/f1000research.93400.r129463

**Published:** 2022-04-11

**Authors:** Nor Azlida Mohd Nor

**Affiliations:** 1 Department of Community Oral Health & Clinical Prevention, Faculty of Dentistry, University of Malaya, Kuala Lumpur, Malaysia

**Abstract:**

- Background section is too long, suggest to shorten it and make it concise. If word count permits, authors may want to expand the results and conclusion sections.

**Methods:**

 Page 3:

- Sample size calculation is not very clear. Authors can add explanation on sample size calculation, and how they get (n=100).

Page 4:

- For a clinical examiner, is there any training and calibration exercise conducted? What is the examiner reliability score? Where is the clinical examination conducted? Is it at the dental school or at the children’s school? Is the examination performed under natural light or portable light?
- The principal researcher performed the child’s oral hygiene examinations using the oral hygiene index simplified (OHI-S) - please add reference.
- Sentences related to description about the index for clinical examination require reference.
- Parental questionnaire - how did you develop the questionnaire? Was the questionnaire validated? There is a lack of clear explanation on the survey instrument used. Authors need to add this information to make it clearer to reader.
- Parents’ knowledge score – how do you assign a score for each question. Is there any reference for the cut-off category for high and low knowledge score?

**Results:**

 Page 5:

- Table 1 caption: suggest change the word “each subject” to “study participants”
- Table 3: The categorisation of good, moderate and poor OHI-S score should also be explained in the methods section.

**Discussion:**

 Page 6:

- Last paragraph - in statistics, the p value is not 0.00 but can be written as  (p<0.001) instead.

Page 7:

- First paragraph - authors can interpret the findings and provide clearer explanation, rather than using statistical jargon.
- Paragraph 4 & 5- how can the authors relate the factors stated in paragraph 4 with the demographic characteristics of the participants in your study?

**Limitation**:

- Do you think the small sample size is part of the study limitation?

**Conclusion**:

- I found the term “contradictory relationship” is rather confusing. Do you mean inverse relationship?

Is the work clearly and accurately presented and does it cite the current literature?

Yes

If applicable, is the statistical analysis and its interpretation appropriate?

Partly

Are all the source data underlying the results available to ensure full reproducibility?

Yes

Is the study design appropriate and is the work technically sound?

Partly

Are the conclusions drawn adequately supported by the results?

Yes

Are sufficient details of methods and analysis provided to allow replication by others?

Partly

Reviewer Expertise:

dental public health

I confirm that I have read this submission and believe that I have an appropriate level of expertise to confirm that it is of an acceptable scientific standard, however I have significant reservations, as outlined above.
